# Cellular succinate metabolism and signaling in inflammation: implications for therapeutic intervention

**DOI:** 10.3389/fimmu.2024.1404441

**Published:** 2024-06-11

**Authors:** Hong Huang, Gejing Li, Yini He, Jing Chen, Jianye Yan, Qin Zhang, Liqing Li, Xiong Cai

**Affiliations:** ^1^ Department of Rheumatology of First Hospital and Institute of Innovation and Applied Research in Chinese Medicine, Hunan University of Chinese Medicine, Changsha, Hunan, China; ^2^ The Central Research Laboratory, Hunan Traditional Chinese Medical College, Zhuzhou, Hunan, China

**Keywords:** succinate, inflammation, hypoxia-inducible factor-1α, succinate receptor 1, therapeutic intervention

## Abstract

Succinate, traditionally viewed as a mere intermediate of the tricarboxylic acid (TCA) cycle, has emerged as a critical mediator in inflammation. Disruptions within the TCA cycle lead to an accumulation of succinate in the mitochondrial matrix. This excess succinate subsequently diffuses into the cytosol and is released into the extracellular space. Elevated cytosolic succinate levels stabilize hypoxia-inducible factor-1α by inhibiting prolyl hydroxylases, which enhances inflammatory responses. Notably, succinate also acts extracellularly as a signaling molecule by engaging succinate receptor 1 on immune cells, thus modulating their pro-inflammatory or anti-inflammatory activities. Alterations in succinate levels have been associated with various inflammatory disorders, including rheumatoid arthritis, inflammatory bowel disease, obesity, and atherosclerosis. These associations are primarily due to exaggerated immune cell responses. Given its central role in inflammation, targeting succinate pathways offers promising therapeutic avenues for these diseases. This paper provides an extensive review of succinate’s involvement in inflammatory processes and highlights potential targets for future research and therapeutic possibilities development.

## 1 Introduction

Inflammation is fundamentally a protective response against chemical, mechanical, or microbial stimuli, typically manifesting as acute and beneficial to the host. However, when unresolved, this process can transition into chronic inflammation (1). A burgeoning area of research focuses on the intersection of metabolic changes and inflammation (2). Central to this discussion is succinate, a tricarboxylic acid (TCA) cycle intermediate primarily found in the mitochondrial matrix. It serves both as a substrate for succinate dehydrogenase (SDH) and as an electron donor in the electron transport chain (ETC) (3).

Interestingly, elevated succinate levels have been detected in the cytoplasm, suggesting its functions extend beyond those classically understood within the mitochondria (4, 5). Recent evidence underscores succinate’s role as a signaling molecule, particularly under hypoxic conditions common in inflammatory environments (6). Here, disruptions in the TCA cycle and respiratory chain lead to cytoplasmic succinate accumulation (7), contributing to several biological processes such as stabilization of hypoxia-inducible factor-1α (HIF-1α), generation of reactive oxygen species (ROS), and protein succinylation (8–10). Intracellular succinate is increasingly recognized as a pro-inflammatory mediator (10).

Moreover, extracellular succinate exerts significant physiological effects by activating succinate receptor 1 (SUCNR1, also known as GPR91), a G protein-coupled receptor on the plasma membrane (11, 12). Given its role as a GPCR, SUCNR1 is a promising target for pharmaceutical modulation using small molecules (13–15). While traditionally implicated in promoting pro-inflammatory responses (16–18), recent findings also suggest anti-inflammatory functions associated with SUCNR1 (19). The precise role of the succinate/SUCNR1 axis in either exacerbating or ameliorating disease remains an area of active investigation, but it is clear that this pathway is crucial in linking metabolism with immune regulation (20, 21). Despite significant advancements in this area, further research is warranted to comprehensively unravel both the intracellular and extracellular functions of succinate.

In this paper, we explore the multifaceted roles of succinate in inflammation, particularly its pivotal function as a signaling molecule within immune responses. We further discuss how these insights inform our understanding of specific diseases, provide opportunities for modulating succinate levels, and pave the way for novel therapeutic interventions.

## 2 Succinate release, accumulation, and transport

Succinate, a central metabolite in the TCA cycle, is produced from α-ketoglutarate (α-KG) through a two-step process. First, α-KG is decarboxylated to succinyl-CoA by α-KG dehydrogenase, followed by conversion to succinate via succinyl-CoA synthetase. This reaction represents a substrate-level phosphorylation, generating GTP (or ATP in some organisms) directly (3). Under normal conditions, succinate is promptly oxidized to fumarate by SDH.

However, under conditions like immune cell activation or tumorigenesis where cells predominantly rely on anaerobic glycolysis, alternative pathways lead to succinate accumulation (7, 22). SDH, which consists of subunits SDHA, SDHB, SDHC, and SDHD, is integral to both the TCA cycle and the mitochondrial ETC as respiratory complex II. It is located within the inner mitochondrial membrane (23) and facilitates the conversion of succinate to fumarate while simultaneously reducing ubiquinone (UQ) to ubiquinol (UQH2) and flavin adenine dinucleotide (FAD) to FADH2—critical steps in ATP production (3, 24). Under hypoxic conditions, SDH activity diminishes, leading to the reversal of electron flow and enabling fumarate to serve as the terminal electron acceptor in the ETC, resulting in succinate accumulation (2, 25). Chouchani et al. reported that sources such as the purine nucleotide cycle and malate/aspartate shuttle contribute fumarate to SDH during ischemia (8), while anaerobic glycolysis primarily produces succinate via glutamine-dependent anaplerosis to α-KG (26). Additionally, succinate can be generated from the γ-aminobutyric acid (GABA) shunt, with the pathway’s activity linked to the expression levels of GABA transporter family members solute carrier 12 (SLC6A12) and SLC6A13 (9). Another significant source of intracellular succinate is the uptake of extracellular succinate by sodium-dependent transporters of the SLC13 family (27).

**Figure 1:**
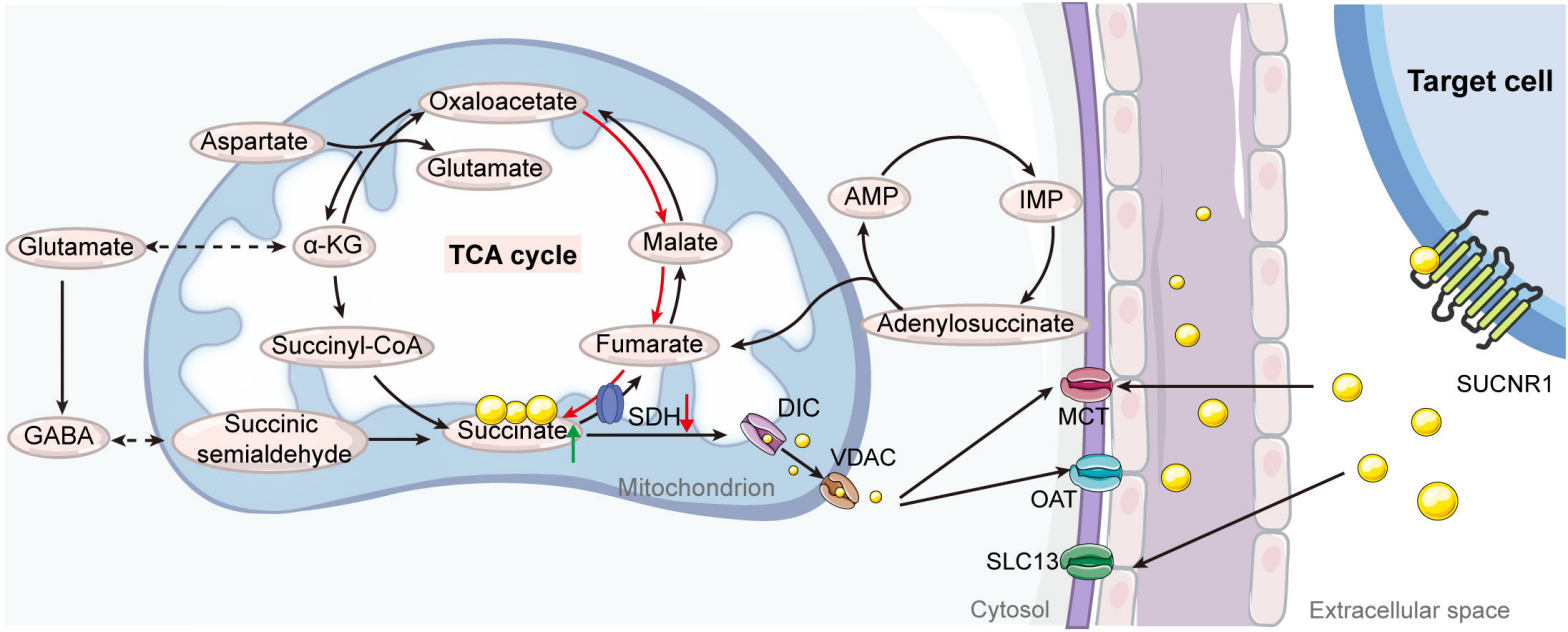
Illustration of the pathways of succinate production and transport. Under normal physiological conditions within the tricarboxylic acid (TCA) cycle, succinate is synthesized as an intermediate from the transformation of succinyl-CoA and is then converted into fumarate by succinate dehydrogenase (SDH). Under specific stress conditions or metabolic alterations, mitochondrial succinate can accumulate through several alternative pathways, including reverse activity of SDH, the purine nucleotide cycle, malate/aspartate shuttle, glutamine-dependent anaplerosis, and the γ-aminobutyric acid (GABA) shunt. When mitochondrial succinate levels exceed cellular requirements, it is transported into the cytosol through mitochondrial dicarboxylate carriers (DICs) and voltage-dependent anion channels (VDACs). The efflux of succinate from the cytosol to the extracellular space is mediated by organic anion transporters (OATs) and monocarboxylate transporters (MCTs). Additionally, extracellular succinate can be reabsorbed via the solute carrier family 13 (SLC13) and further facilitated by MCTs. AMP, adenosine monophosphate; IMP, inosine monophosphate.

Given its charged nature at physiological pH, succinate’s membrane permeability is naturally restricted, necessitating specialized transporters for its movement from the mitochondrial matrix to the cytosol and across the plasma membrane into the extracellular space (28). Intracellularly, the dicarboxylate carrier (DIC), a member of the SLC25 transporter family, transports accumulated succinate across the mitochondrial inner membrane. The voltage-dependent anion channel (VDAC) facilitates this process at the outer membrane (29, 30). Once in the cytosol, succinate is exported to the extracellular environment via organic anion/dicarboxylate transporters (OATs) (27). Conditions of increased energy demand trigger anaerobic energy systems, leading to excessive lactate production and cellular acidification (31, 32). This acidification modifies succinate’s chemical structure, allowing it to cross cell membranes with assistance from plasma membrane monocarboxylate transporter 1 (MCT1) (33, 34). Additionally, Gudgeon et al. (35) observed that succinate uptake in CD4^+^ T cells is partially dependent on MCT1. In macrophages, enhancing transporter-mediated succinate uptake is crucial for augmenting and sustaining inflammatory responses (27). The key role of these succinate transporters lies in exporting succinate to different cellular compartments, where it serves as a signaling molecule (10). Targeting these transporters offers a potential strategy to regulate extracellular succinate levels, indirectly impacting SUCNR1 activation (**Figure 1**).

## 3 Succinate and inflammation: an intricate nexus

Succinate, increasingly recognized as a signaling molecule, plays a pivotal role at the juncture of metabolism and inflammation (36). It serves as a crucial mediator linking intracellular and extracellular inflammatory signaling and regulates inflammation through various distinct mechanisms (37). This multifaceted relationship not only deepens our understanding of cellular responses during inflammation but also highlights potential therapeutic targets in inflammatory diseases.

### 3.1 Intracellular succinate, HIF-1α stabilization and inflammation

HIF-1α, a key transcription factor, regulates cellular responses to hypoxia. Upon stabilization, HIF-1α translocates into the nucleus, binding to hypoxia-responsive elements to initiate transcription of genes (38), including those related to inflammation such as interleukin-1β (IL-1β) (9, 39). Prolyl hydroxylases (PHDs), part of the α-KG-dependent dioxygenase family, are integral to cellular oxygen sensing and regulate HIF-1α stability. An increase in cytoplasmic succinate, following PHD inhibition, activates HIF-1α, thereby amplifying the transcription of pro-inflammatory genes (40). Although this activation is beneficial for cellular adaptation to hypoxic conditions, excessive succinate can induce a “pseudohypoxia” state (41, 42), enhancing HIF-1α activity even in oxygen-rich environments, a condition frequently observed in tumors with mutated SDH (43). Consequently, inhibiting PHDs hinders HIF-1α degradation even in oxygen-rich environments, hence triggering the activation of a spectrum of target genes (41).

**Figure 2:**
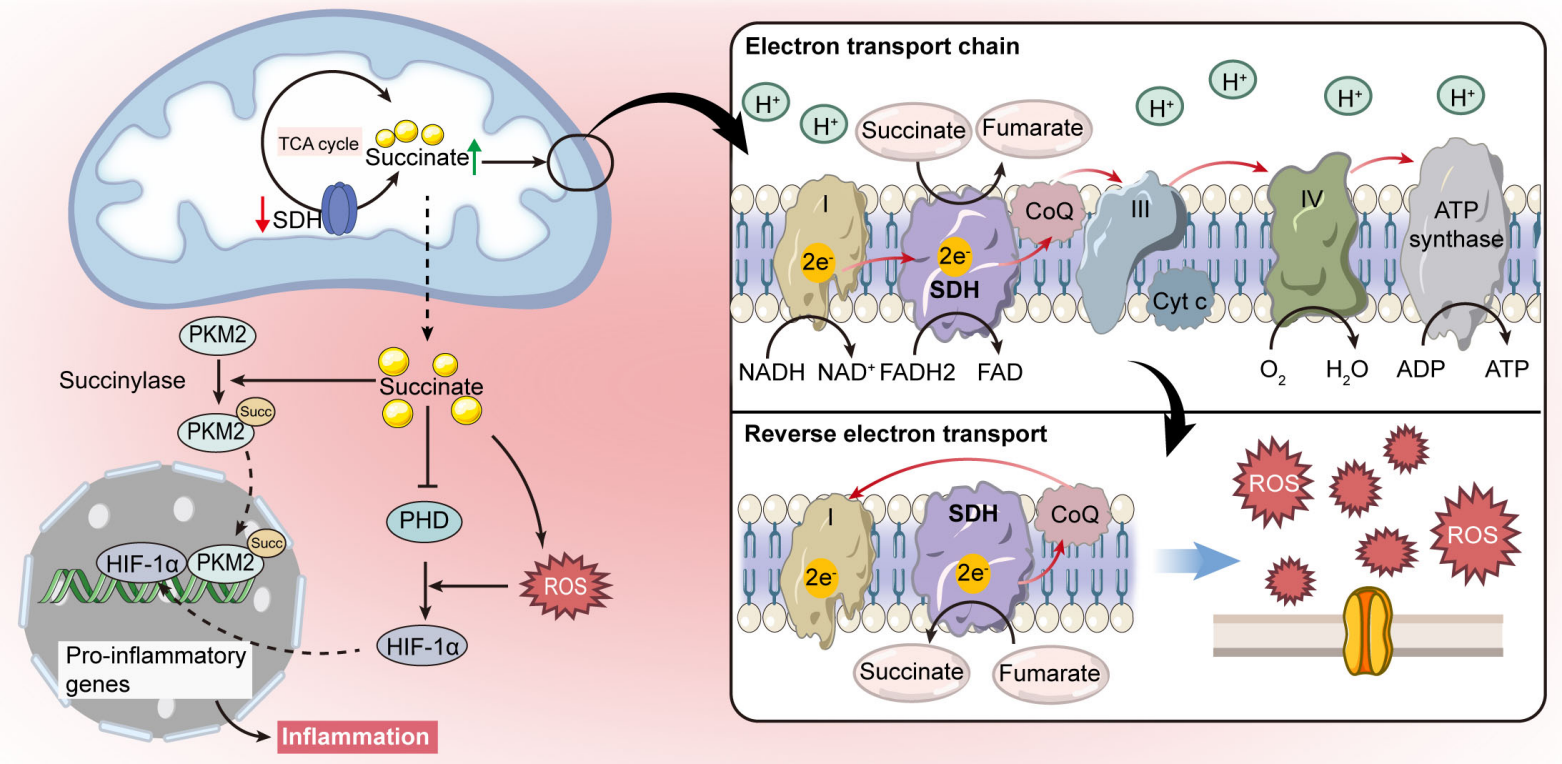
Intracellular signaling pathways of succinate. Succinate acts as a signal molecule that stabilizes hypoxia-inducible factor-1α (HIF-1α) by inhibiting prolyl hydroxylase (PHD), which leads to the production of pro-inflammatory cytokines. Succinate can also induce redox signaling by promoting mitochondrial reactive oxygen species (ROS) production through reverse electron transport (RET), further supporting HIF-1α stabilization. Moreover, succinate accumulation is linked to succinylation, which regulates metabolic enzyme activities.

Additionally, succinate has been shown to indirectly stabilize HIF-1α by inducing the production of ROS (44, 45). ROS mediates the oxidation of Fe^2+^ to Fe^3+^, inhibiting PHD activity that relies on Fe^2+^. This inhibition leads to the activation of HIF-1α (46). In macrophages activated by lipopolysaccharide (LPS), elevated succinate levels enhance UQ reduction, leading to “reverse electron transport (RET)” which channels electrons back to respiratory complex I (47, 48). This process significantly increases ROS production, further stabilizing HIF-1α through PHD inhibition (40) (**Figure 2**). As a result, high levels of succinate not only disrupt ATP synthesis and oxidative phosphorylation but also enhance ROS generation, amplifying pro-inflammatory signals (49, 50).

Moreover, increased cytoplasmic succinate levels promote post-translational modifications, such as succinylation, which regulates the activity of metabolic enzymes in mitochondria (51–53). In LPS-activated macrophages, a metabolic shift occurs from reliance on mitochondrial ATP production to dependence on aerobic glycolysis, with pyruvate kinase M2 (PKM2) playing a critical role (54). Hypersuccinylation of PKM2 facilitates its nuclear translocation, promoting the formation of a PKM2-HIF-1α complex at the IL-1β gene promoter, as illustrated in **Figure 2**. This interaction intensifies the severity of dextran sulfate sodium (DSS)-induced colitis in mice (54, 55). Additionally, the sirtuin family enzyme SIRT5 is implicated in modulating PKM2 succinylation and its enzymatic activity, thus potentially attenuating pro-inflammatory responses in macrophages (54).

### 3.2 Extracellular succinate and SUCNR1 signaling in inflammation

Upon release from cells, succinate interacts with its membrane receptor, SUCNR1, which is sensitively responsive to extracellular succinate levels (56). SUCNR1 is expressed across a variety of tissues, including the kidney, small intestine, liver, spleen, retina, and heart, as well as within specific immune cells such as macrophages, dendritic cells (DCs), and some T-cell subsets. This wide distribution underscores its significant role in modulating immune responses (17, 28, 57). The expression of SUCNR1 is influenced by the extracellular concentration of succinate, which under normal physiological conditions in plasma and urine remains below the activation threshold of the receptor (58). However, these concentrations are approximately twofold lower than the EC_50_ for SUCNR1, indicating that a substantial increase in succinate levels is necessary to fully engage the receptor (59). The signaling pathways activated by SUCNR1 involve two G proteins—Gq and Gi (11, 60). Activation of protein kinase C (PKC) and mitogen-activated protein kinase (MAPK) cascades, along with calcium mobilization, are mediated through the Gq pathway, whereas the inhibition of cyclic adenosine monophosphate (cAMP) is facilitated by the Gi pathway (12). These mechanisms highlight the critical role of the succinate-SUCNR1 axis in the regulation of inflammatory processes.

While intracellular succinate is acknowledged as a necessary pro-inflammatory signal, its extracellular presence not only amplifies pro-inflammatory responses but, under certain conditions, can also exert anti-inflammatory effects (27). In induced neural stem cells (iNSCs) and somatic neural stem cells (NSCs) that utilized to mimic the inflammatory cascade, Peruzzotti-Jametti et al. showed that succinate induces an anti-inflammatory response through SUCNR1 signaling, leading to the release of prostaglandin E2 (PGE2), which modulates various cell types within the inflammatory microenvironment. Additionally, the absence of SUCNR1 in NSCs significantly reduces anti-inflammatory actions both *in vitro* and *in vivo (* 61). Succinate has been proposed as a potential therapeutic for severe sepsis, primarily by enhancing ATP generation, which is crucial for rescuing tissues from the bioenergetic dysfunction associated with sepsis (62). Recent research suggests that activating SUCNR1 in macrophages within adipose tissue could initiate an anti-inflammatory response (63). Moreover, succinate may inhibit the release of inflammatory mediators like IL-6, IL-1β, nitric oxide (NO), and tumor necrosis factor (TNF) in inflammatory macrophages, independently of SUCNR1 signaling (64). However, succinate can also exacerbate inflammatory conditions by enhancing oxidative stress or driving inflammation, as observed in macrophages exposed to LPS, in mouse models of antigen-induced arthritis, and 2,4,6-trinitrobenzene sulfonic acid (TNBS)-induced colitis (9, 16, 65). The role of succinate in inflammation and its broader physiological and pathological implications are increasingly focused on the dynamics of its receptor, SUCNR1 (66, 67). The dual pro- and anti-inflammatory capacities of succinate during inflammation appear to be highly context-dependent.

### 3.3 Cellular succinate immunometabolism

#### 3.3.1 Succinate and macrophages

Macrophages are heterogeneous immune cells that play a crucial role in immune responses. Upon classical activation by the toll-like receptor 4 (TLR4) ligand LPS, macrophages transition from ATP production via oxidative phosphorylation to glycolysis, which is accompanied by an increase in succinate levels (68). This metabolic shift in macrophages is significant, as cancer cells have also been observed to release succinate into the extracellular space, thereby facilitating the polarization of macrophages into tumor-associated macrophages through SUCNR1 (67). Succinate serves not only as a marker of pro-inflammatory activity but also contributes to the pro-inflammatory capabilities of macrophages. In the inflammatory microenvironment, excessive pro-inflammatory activity can exacerbate conditions such as inflammatory bowel disease (IBD) (69, 70). Moreover, succinate acts as a chemokine, inducing cell migration in the U937 macrophage cell line via SUCNR1 activation (17). Gobelli et al. underscored the essential roles of SDHA and SDHB in LPS-mediated production of IL-10 and IL-1β, HIF-1α stabilization, and tyrosine phosphorylation of the transcription factor signal transducer and activator of transcription 3 (STAT3) in macrophages. However, they noted that these components are not necessary for the production of IL-6 and TNF-α (71).

**Figure 3:**
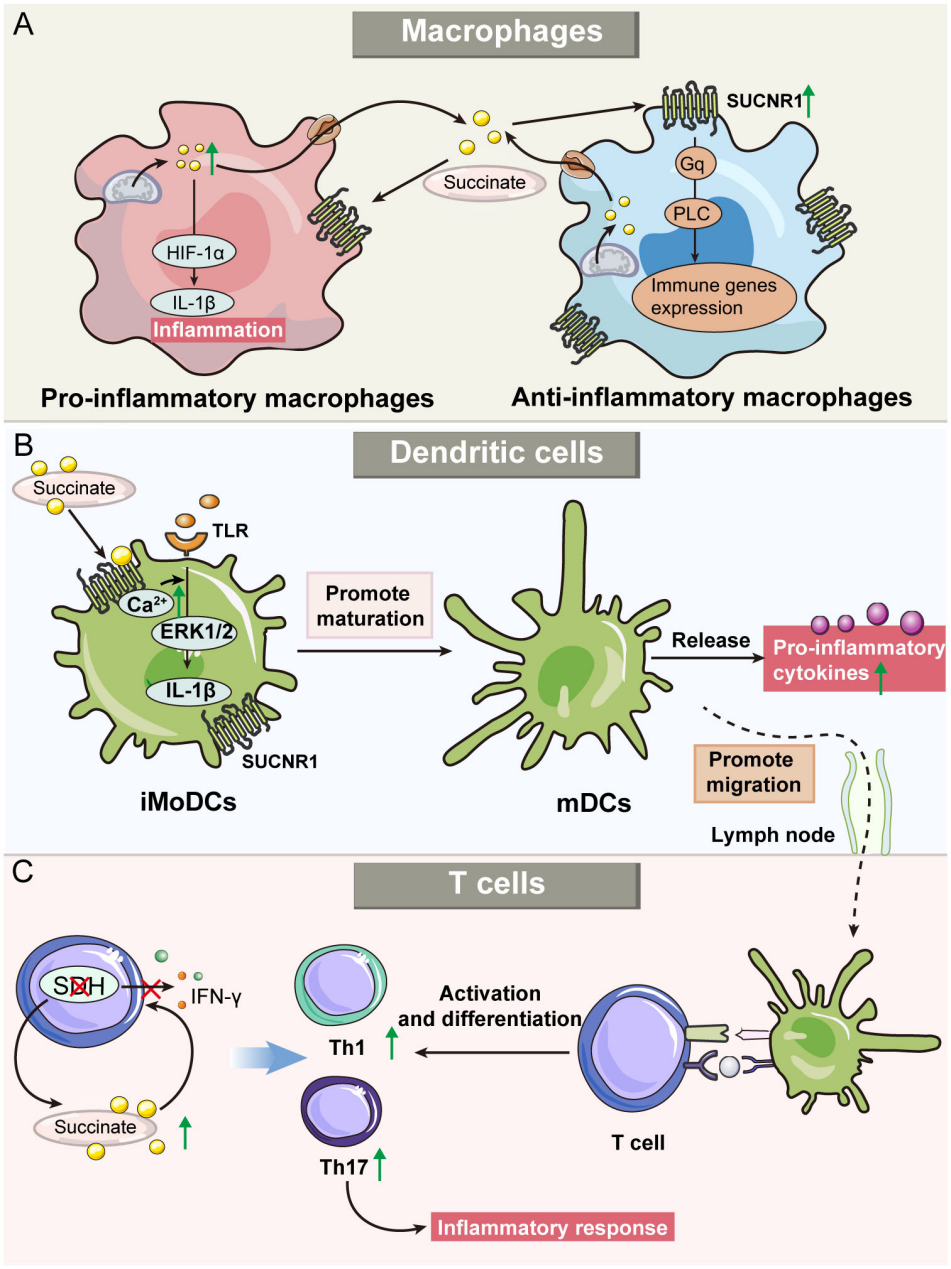
Roles of succinate and SUCNR1 in immune cells, including macrophages, dendritic cells (DCs), and T cells. **(A)** Succinate modulates the activities of both pro-and anti-inflammatory macrophages. Succinate serves as an indicator of pro-inflammatory activity, which promotes the production of IL-1β in macrophages and triggers inflammatory response. Furthermore, succinate also enhances the activity of anti-inflammatory macrophages through the SUCNR1-mediated Gq pathway. **(B)** Exogenous succinate regulates the function of DCs. Immature monocyte-derived DC (iMoDCs) express high levels of SUCNR1, and succinate promotes iMoDC maturation. In addition, succinate can upregulate the migration of mature DCs into the lymph nodes and enhance antigen presentation by DCs. **(C)** Succinate’s role in T cell inflammatory responses. Succinate dehydrogenase (SDH) deficiency leads to increased succinate, which regulates cytokine secretion, alters T-cell metabolism and promotes differentiation into Th1 and Th17, thereby regulating the inflammatory response.

The succinate-SUCNR1 axis, while recognized for inducing and exacerbating inflammation, also plays a crucial role in polarizing anti-inflammatory macrophages and regulating inflammation (72, 73). Intriguingly, although succinate is produced by pro-inflammatory macrophages, its receptor SUCNR1 is primarily found in anti-inflammatory macrophages (63, 74). Recent studies have highlighted its role in the inflammatory program, particularly in promoting anti-inflammatory activity through SUCNR1 (63). Park et al. demonstrated that macrophages treated with succinate could mitigate colitis (75). Specifically, adipose-derived mesenchymal stem cells (ADMSCs) were stimulated by intracellular succinate accumulation to secrete PGE2, a soluble molecule that reduced inflammation in macrophages and shifted their phenotype from pro-inflammtory to anti-inflammatory macrophages (76). Furthermore, extracellular succinate enhances this change by binding to SUCNR1 on anti-inflammatory macrophages via Gq and PLC pathway signaling (77). Notably, research indicates that a higher α-KG/succinate ratio favors anti-inflammatory macrophage activation, whereas a lower ratio promotes pro-inflammatory activation (78). This finding suggests that while succinate can promote anti-inflammatory macrophage polarization, the precise balance between its concentration and that of other metabolites is critical in determining macrophage polarization direction. Additional research is necessary to ascertain the specific concentration thresholds of succinate that influence this polarization process (**Figure 3A**).

#### 3.3.2 Succinate and DCs

DCs are crucial antigen-presenting cells that play a vital role in regulating the adaptive immune system. During inflammation, DCs capture antigens, mature, and migrate to lymphoid tissues to present these antigens to naive T cells. This process is accompanied by the secretion of a range of pro-inflammatory cytokines, further amplifying the immune response (79). In immature monocyte-derived DCs (iMoDCs), elevated expression of SUCNR1 was observed, which decreased as the cells matured (17). The binding of extracellular succinate to SUCNR1 on iMoDCs triggers the mobilization of intracellular calcium and phosphorylation of extracellular signal-regulated kinases 1 and 2 (ERK1/2). This activity, in synergy with TLRs, promotes iMoDC maturation and secretion of IL-1β, thus enhancing the inflammatory response to pathogens. Additionally, succinate significantly increases the migratory capacity of DCs and their ability to activate T cells in an antigen-specific manner. Notably, these succinate-mediated effects are substantially reduced in the absence of SUCNR1, as demonstrated in knockout studies (17). Mice treated with succinate developed more severe arthritis, characterized by an increased presence of T helper cell 17 (Th17) cells and SUCNR1-expressing DCs in lymph nodes (80). Importantly, HIF-1α has been identified as a crucial mediator in the regulatory effects of DCs in inflammatory diseases. For example, in a colitis mouse model, the absence of HIF-1α in intestinal DCs exacerbated colitis symptoms (81). Similarly, in obesity models, HIF-1α deficiency in adipose tissue DCs led to enhanced inflammation (82) (**Figure 3B**).

#### 3.3.3 Succinate and T cells

Succinate not only indirectly modulates T-cell function through its effects on DCs but also exerts a direct regulatory impact within the inflammatory microenvironment. It has been observed that succinate can inhibit degranulation and cytokine secretion in CD4^+^ and CD8^+^ T cells, particularly affecting the production of interferon-γ (IFN-γ) (35). Additionally, inhibiting SDH during T-cell activation significantly impairs the expression of activation markers, production of inflammatory cytokines, and cell proliferation (83). Conversely, Chen et al. reported that SDH dysfunction, while hindering cell proliferation, actually enhances inflammatory responses in T cells. Specifically, T cells deficient in SDHB exhibited an elevated succinate/α-KG ratio, upregulation of pro-inflammatory gene markers, and a propensity to differentiate into pro-inflammatory Th1 and Th17 lineages (84). In experimental autoimmune uveitis in mice, succinate was shown to enhance the formation of neutrophil extracellular traps and the frequencies of Th1/Th17 cells, accompanied by increased production of IFN-γ and IL-17A through the succinate-SUCNR1 axis (85). These findings highlight the pivotal roles of SDH and succinate in the adaptive immune system (**Figure 3C**).

## 4 Dysregulated succinate metabolism and disease

**Figure 4:**
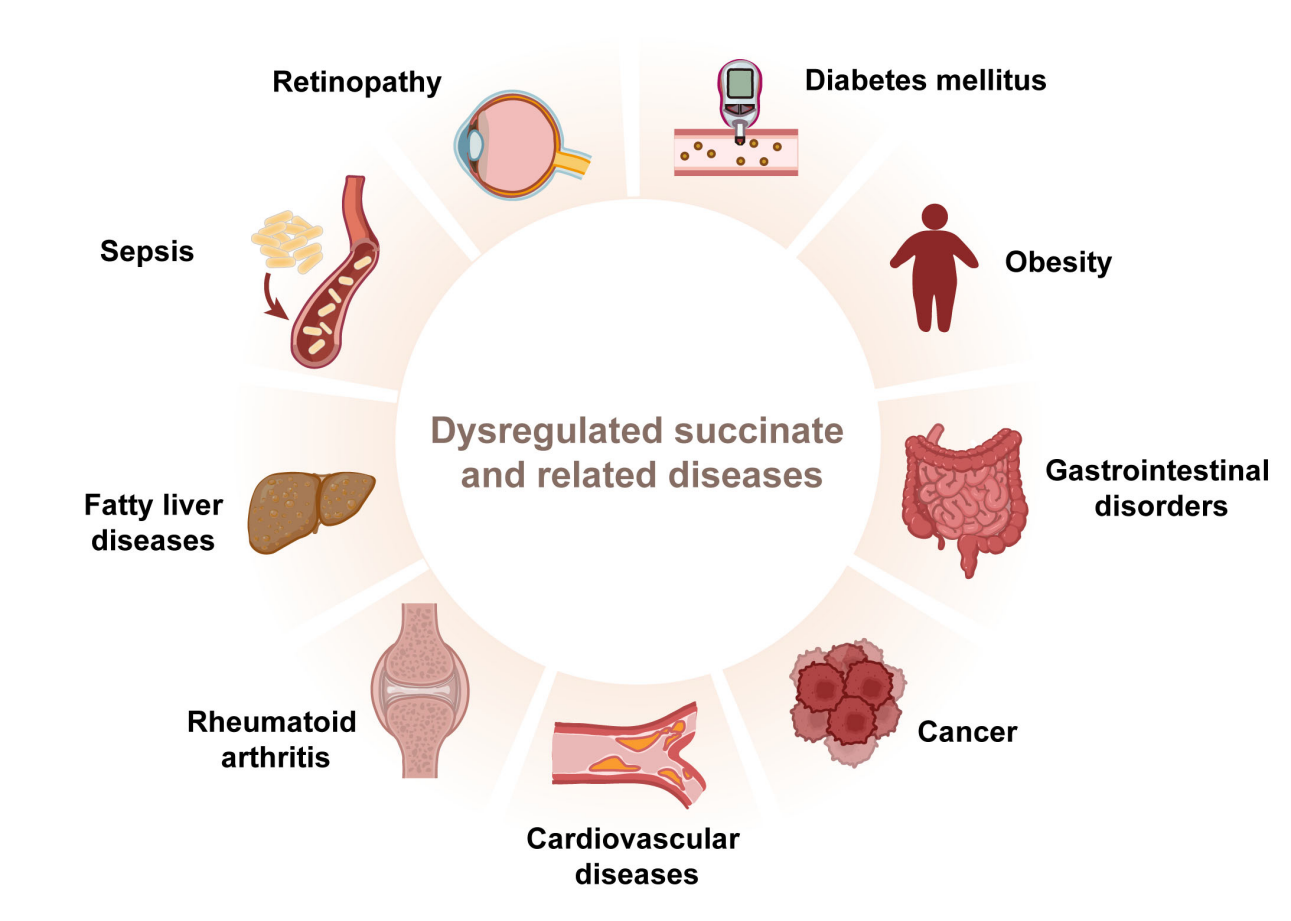
Dysregulated succinate contributes to various diseases. Succinate plays an important role in inflammatory diseases.

Succinate’s functions extend well beyond its role as a mere metabolic intermediate; it influences blood pressure, the renin-angiotensin system in the kidney, lipolysis in adipose tissue, skeletal muscle remodeling, and retinal vascularization, affecting the pathophysiology of a broad spectrum of diseases (11, 19). Dysregulation of succinate metabolism or its accumulation has been linked to various disorders ranging from metabolic syndromes to inflammatory diseases, including gastrointestinal disorders (58), diabetes mellitus (86), retinal complications (87), cardiovascular disease (88–90), fatty liver diseases (19), rheumatoid arthritis (91), sepsis (92), obesity (93), and some cancers (94) (**Figure 4**). Representative examples will be further discussed in the following section (**Table 1**).

**Table 1:** Evidences of dysregulated succinate and its effects in diseases.

Disease	Changes of succinate	Effect	Mechanism
Rheumatoid arthritis	Succinate is accumulated in the synovial fluid	Promotes inflammation	1.Triggers the NLRP3 inflammasome and the secretion IL-1β 2.Stimulates the release of VEGF from endothelial cells, which leads to angiogenesis. 3.Promotes the proliferation of Th17 cells
Inflammatory bowel disease	Elevated levels of succinate in serum, plasma, and intestinal, and enhanced expression of SUCNR1 in the intestinal tissues of IBD patients	Promotes inflammation	1.Triggers fibroblast activation 2.Stimulates pro-inflammatory responses in macrophages
Obesity	Circulating succinate was significantly increased in both rodent models and patients with obesity	A dual role in regulating inflammation	1.Promotes macrophage infiltration into adipose tissue 2.Promotes type 2 immunity 3.Facilitates the development of barrier-promoting goblet cells
Atherosclerosis	Elevated levels of succinate	A mediator in driving the progression of atherosclerotic lesions	1.Promotes smooth muscle cell phenotype transformation 2.Promotes macrophage polarization and endothelial cell dysfunction

### 4.1 Rheumatoid arthritis

Rheumatoid arthritis, a chronic inflammatory disorder, is closely associated with metabolic dysregulation, which influences its progression and symptom severity (95, 96). Succinate levels are notably high in the synovial fluid of both antigen-induced arthritis mice and patients with rheumatoid arthritis (16, 97). Metabolic profiling has identified succinate as a distinctive metabolite differentiating rheumatoid arthritis from other arthropathies (98). This accumulation triggers macrophages in the synovial fluid to release IL-1β, mediated through SUCNR1 signaling (16). Additionally, succinate in synovial fibroblasts activates the NOD-, LRR-, and pyrin domain-containing protein 3 (NLRP3) inflammasome, enhancing IL-1β secretion (99). Treatment of endothelial cells with LPS and exposure of these cells along with synovial fibroblasts to hypoxic conditions result in succinate accumulation, which induces angiogenesis through the release of vascular endothelial growth factor (VEGF), associated with HIF-1α stabilization and SUCNR1 activation by extracellular succinate (91). This endothelial activation and increased angiogenesis contribute to the pathogenesis of rheumatoid arthritis by facilitating leukocyte recruitment and migration and promoting hyperplasia in the synovial pannus (100). Moreover, SUCNR1 functions as a chemotactic gradient sensor, orchestrating DC migration toward lymph nodes, thereby leading to the proliferation of Th17 cells. These cells are closely associated with articular lesions and are known to intensify inflammation, neutrophil infiltration, mechanical hyperalgesia, and bone erosion within joints (80). These findings underscore the critical role of succinate in regulating inflammation in rheumatoid arthritis (101).

### 4.2 Inflammatory bowel disease

IBD, encompassing two primary phenotypes, Crohn’s disease (CD) and ulcerative colitis (UC), is a chronic disorder characterized by recurrent inflammation of the gastrointestinal tract (102). Studies have shown increased levels of succinate in the sera, plasma, and intestinal tissues of IBD patients, along with enhanced expression of SUCNR1 in these tissues (65, 103, 104). This is supported by findings in mice, where those colonized with fecal samples from CD and UC patients displayed higher fecal succinate levels compared to those colonized from healthy individuals, indicating that succinate accumulation is a metabolic hallmark of IBD-associated dysbiosis (105). SUCNR1 not only triggers fibroblast activation and stimulates pro-inflammatory responses in macrophages but also contributes to colitis and intestinal fibrosis in murine models (65). Furthermore, a relationship has been reported between increased succinate levels in the gut lumen and microbiome dysbiosis in both IBD patients and animal models of intestinal inflammation (30, 106).

### 4.3 Obesity

Recent studies indicate that circulating succinate levels are significantly elevated in both rodent models and patients with obesity (93, 107). The inflammation of adipose tissue, a hallmark of obesity, drives insulin resistance and subsequently, diabetes mellitus. Succinate and its receptor SUCNR1 exacerbate this obesity-induced inflammation, promoting the infiltration of macrophages into adipose tissue, which is a key contributor to the glucose intolerance observed in type 2 diabetes (21). Interestingly, the effects of SUCNR1 deficiency in macrophages vary depending on their location and the level of inflammation. In subcutaneous fat, SUCNR1-deficient macrophages tend to exhibit a pro-inflammatory phenotype, unlike their wild-type counterparts. Conversely, in visceral fat, which is typically pro-inflammatory, macrophages show reduced inflammation when SUCNR1 is deficient (63). Previous studies have demonstrated that the succinate-SUCNR1 axis plays a crucial role in promoting type 2 immunity in the gut, acting as an essential anti-inflammatory regulator (108). While obesity is associated with increased succinate levels (93), it often features impaired SUCNR1 signaling, leading to what is described as a “succinate-resistant state” (63). In SUCNR1-deficient mouse models, succinate-SUCNR1 signaling initiates type 2 immune responses in the intestine, facilitates the development of barrier-promoting goblet cells, mitigates high-fat diet (HFD)-induced mucosal barrier impairments and intestinal dysbiosis, and ultimately exhibits anti-obesity effects. This research highlights the protective function of the succinate-SUCNR1 axis against intestinal barrier dysfunction induced by HFD (109).

### 4.4 Atherosclerosis

Atherosclerosis is a multifactorial pathological condition characterized by vascular inflammation and the accumulation of lipids and fibrous elements in the vessel walls (110). Elevated levels of succinate may act as a mediator driving the progression of atherosclerotic lesions by activating the SUCNR1 pathway, which results in phenotypical transformations of smooth muscle cells, macrophage polarization, and endothelial cell dysfunction (111). Studies have shown that patients with coronary heart disease exhibit notably higher levels of succinate and IL-1β compared with healthy individuals, with a positive correlation between succinate and IL-1β levels. The glycolytic metabolism triggered by the release of succinate is evident in atherosclerotic pathology. This, in turn, stimulates the succinate/IL-1β signaling pathway through SUCNR1, thereby exacerbating inflammatory responses (112).

## 5 Pharmacological interventions targeting succinate metabolism

Targeting succinate metabolism is emerging as a promising approach for the therapeutic management of various diseases. This section highlights pharmacological strategies that intervene in succinate metabolism and discusses their potential implications for therapeutic applications.

### 5.1 Enzyme inhibitors targeting succinate production and metabolism

In the metabolism of pro-inflammatory macrophages, Mills et al. confirmed the critical role of mitochondrial SDH in regulating LPS-induced gene expression. Specifically, elevated succinate oxidation via active SDH increases the expression of inflammatory genes, while inhibition of SDH fosters an anti-inflammatory phenotype (113). Malonate, a competitive inhibitor at the carboxylate site of SDH, and its cell-permeable prodrug dimethyl malonate (DMM), which produces malonate intracellularly, lead to succinate accumulation. DMM has been shown to effectively suppress the expression of pro-inflammatory genes, such as IL-1β and HIF-1α, and simultaneously enhance the expression of anti-inflammatory genes like IL-1RA and type I IFN-inducible genes (113). Interestingly, DMM does not affect TNF-α levels, suggesting a specific pathway of action (113). In conditions like cellular hypoxia in synovial fibroblasts and ischemia, SDH is proposed to function in reverse, converting fumarate to succinate (8). DMM exhibits anti-inflammatory effects by inhibiting the HIF-1α/VEGF axis in hypoxia-treated synovial fibroblasts, preventing succinate accumulation, and inhibiting angiogenesis (91). Moreover, DMM reduces succinate accumulation during ischemia and controls oxidation during reperfusion, thereby decreasing mitochondrial ROS generation and attenuating ischemia-reperfusion injury (8). The malonate ester prodrug diacetoxymethyl malonate shows greater potency than DMM, owing to its accelerated esterase hydrolysis (114). Although succinate accumulates via different mechanisms in LPS-treated macrophages and other conditions, inhibition of SDH similarly reduces IL-1β production (91).

**Figure 5:**
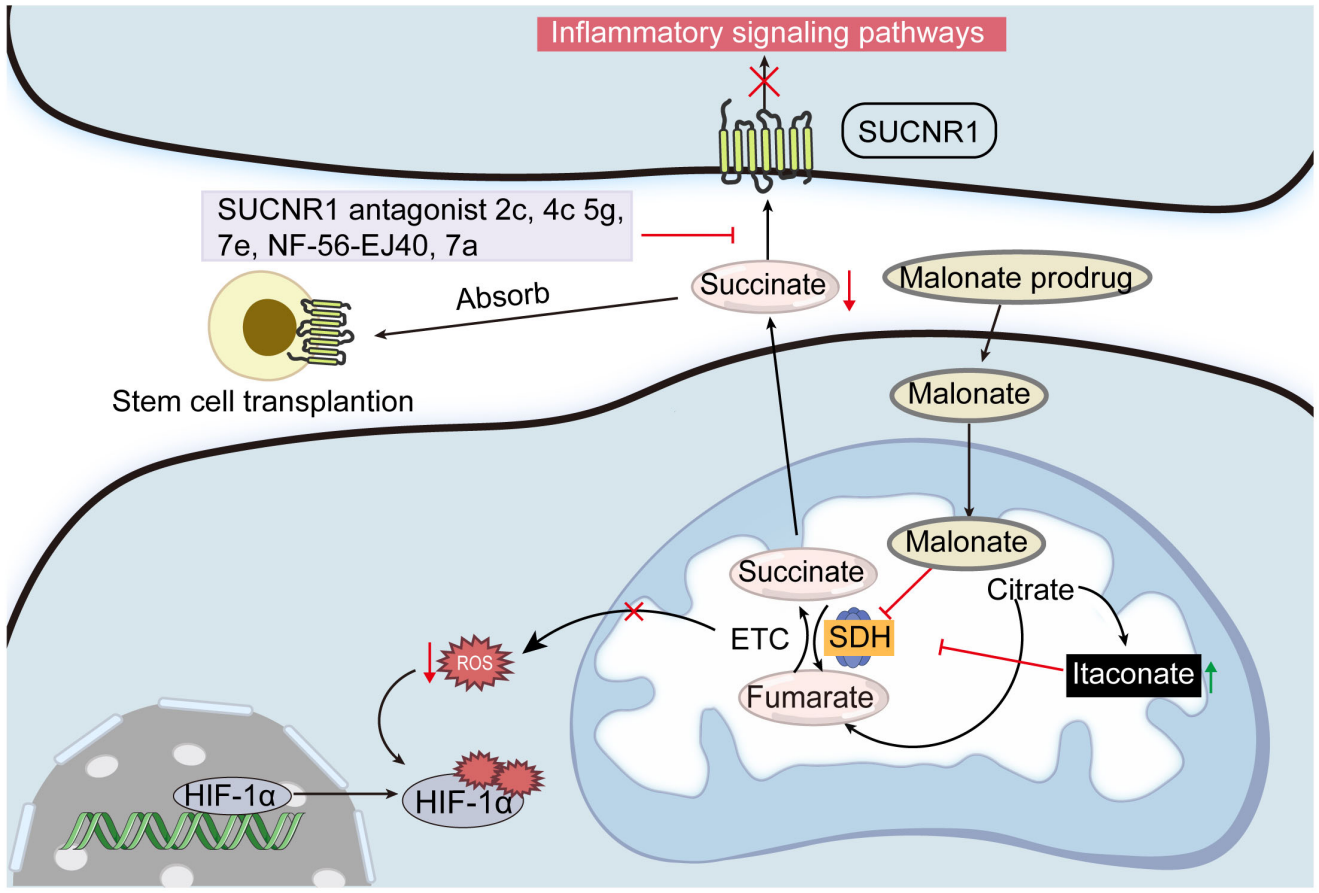
Potential strategies for controlling succinate concentrations. Inhibiting SDH activity with malonate and itaconate is one way to regulate the succinate production in mitochondria. Blocking SUCNR1 activity with its antagonists 2c, 4c, 5g, 7e, and NF-56-EJ40 may reduce the negative effects of excessive succinate accumulation. Furthermore, excessive succinate can be absorbed by transplanting stem cells that express SUCNR.

Itaconate, an endogenous small molecule synthesized by the enzyme cis-aconitate decarboxylase, acts as an immunomodulator and inhibits SDH, impacting metabolic and inflammatory processes in cells (115). Due to its structural similarity to succinate, itaconate was identified decades ago as a competitive inhibitor of SDH. This inhibition leads to elevated succinate accumulation and reduced mitochondrial ROS production, affecting mitochondrial respiratory chain complex I (116). Itaconate demonstrates anti-inflammatory properties *in vitro* and *in vivo* by modulating SDH levels, mitochondrial respiration, and cytokine production (IL-1β, IL-18, IL-6, IL-12, NO, and HIF-1α, but not TNF-α), particularly during macrophage activation and ischemia-reperfusion injury (117). Further studies show that SDH inhibition by itaconate also mitigates cerebral ischemia-reperfusion injury (118, 119). As an SDH inhibitor, itaconate manages oxidative stress and enhances physiological outcomes associated with ischemia-reperfusion injury (120). Research has revealed that both endogenous (117) and exogenous (121) itaconate-mediated SDH inhibition affects mitochondrial function. Importantly, while targeting succinate through this pathway offers valuable insights into its physiological roles, the therapeutic application must be approached cautiously, given the pivotal role of SDH in cellular energetics (**Figure 5**).

### 5.2 Targeting succinate receptor

In recent years, there has been increasing attention on the role of SUCNR1 in various pathophysiological processes, significantly highlighting its potential as a therapeutic target (122). The accumulation of succinate in various inflammatory states further underscores the importance of targeting SUCNR1 to mitigate the detrimental effects of excess succinate (28). Advances in understanding the structure-function relationship of SUCNR1 have facilitated the development of structure-based antagonists (123). Systematic structure-activity relationship studies have identified compounds “2c” and “4c” as effective SUCNR1 antagonists both *in vitro* and *in vivo*. Moreover, compounds “5g” and “7e”, which are structurally distinct and orally bioavailable, have shown potential as promising leads in drug development (13). NF-56-EJ40, a high-affinity SUCNR1 antagonist, effectively reduces succinate/IL-1β signaling in macrophages and HUVECs, demonstrating a potent inhibitory effect (112, 124). Additionally, NF-56-EJ40 substantially attenuates succinate-mediated gene expression in primary human anti-inflammatory macrophages (77). A topically applied gel formulation of the SUCNR1 antagonist compound “7a” has been shown to reduce inflammatory events and osteoclastogenesis *in vivo (* 125). The therapeutic efficacy of “7a” is directly attributed to the suppression of SUCNR1-mediated succinate signaling (126).

Furthermore, the transplantation of cells expressing SUCNR1 into an inflammatory environment is also a promising strategy to mitigate excess succinate. Mesenchymal stem cell (MSC) transplantation, known for its ability to differentiate into various cell types, is utilized to facilitate the repair and regeneration of damaged tissues (127). This method has been demonstrated to effectively decrease succinate levels in tissues, subsequently triggering beneficial effects in adjacent areas. Specifically, upon transplantation, adipose-derived MSCs absorb succinate, decreasing its accumulation and shifting macrophage to pro-inflammatory macrophages, which mitigates DSS-induced colitis in mice (76). Similarly, when transplanting NSCs into the cerebrospinal fluid of mice with experimental autoimmune encephalomyelitis, succinate secreted by type 1 mononuclear phagocytes binds to SUCNR1 on NSCs, thereby reducing the production of succinate in the cerebrospinal fluid and preventing the progression of neuroinflammation (61).

However, the critical involvement of SUCNR1 in essential physiological processes and its significance in regulating energy homeostasis must be carefully considered. The systemic application of SUCNR1 antagonists could lead to severe adverse effects due to the disruption of these essential functions. Additionally, while antagonists serve to inhibit receptor function, there might be scenarios where activating SUCNR1 could offer therapeutic benefits. The challenge lies in employing SUCNR1 antagonists effectively in the treatment of SUCNR1-mediated diseases without compromising its normal or advantageous functions (73).

## 6 Concluding remarks and future perspectives

The correlation between aberrant succinate changes and inflammatory diseases due to overreactive immune responses has been increasingly recognized. The significance of this research lies in its potential to advance the understanding and treatment of inflammatory diseases. Overall, the study of succinate presents a combination of challenges and opportunities, as the delicate balance between the beneficial and detrimental effects of succinate and its receptor is not yet fully understood. As research delves deeper into its complexities, one aspect remains clear: succinate will continue to be a pivotal focus in biomedical research.

## Author contributions

HH: Conceptualization, Project administration, Writing – original draft. GL: Project administration, Writing – review & editing. YH: Project administration, Writing – review & editing. JC: Project administration, Writing – review & editing. JY: Formal analysis, Methodology, Writing – review & editing. QZ: Conceptualization, Project administration, Writing – review & editing. LL: Conceptualization, Project administration, Writing – review & editing. XC: Conceptualization, Project administration, Writing – review & editing.
